# Persisting right-sided chylothorax in a patient with chronic lymphocytic leukemia: a case report

**DOI:** 10.1186/1752-1947-5-492

**Published:** 2011-10-03

**Authors:** Godehard A Scholz, Horia Sirbu, Sabine Semrau, Katharina Anders, Andreas Mackensen, Bernd M Spriewald

**Affiliations:** 1 Department of Internal Medicine 5 - Hematology/Oncology, University of Erlangen-Nürnberg, Krankenhausstrasse 12, 91054 Erlangen, Germany; 2 Department of Thoracic Surgery, University of Erlangen-Nürnberg, Krankenhausstrasse 12, 91054 Erlangen, Germany; 3 Department of Radiation Oncology, University of Erlangen-Nürnberg, Krankenhausstrasse 12, 91054 Erlangen, Germany; 4 Department of Radiology, University of Erlangen-Nürnberg, Krankenhausstrasse 12, 91054 Erlangen, Germany

## Abstract

**Introduction:**

Chylothorax caused by chronic lymphocytic leukemia is very rare and the best therapeutic approach, especially the role of modern immunochemotherapy, is not yet defined.

**Case presentation:**

We present the case of a 65-year-old male Caucasian patient with right-sided chylothorax caused by a concomitantly diagnosed chronic lymphocytic leukemia. As first-line treatment four cycles of an immunochemotherapy, consisting of fludarabine, cyclophosphamide and rituximab were administered. In addition, our patient received total parenteral nutrition for the first two weeks of treatment. Despite the very good clinical response of the lymphoma to treatment, the chylothorax persisted and percutaneous radiotherapy of the thoracic duct was applied. However, eight weeks after the radiotherapy the chylothorax still persisted and our patient agreed to a surgical intervention. A ligation of the thoracic duct via a muscle sparing thoracotomy was performed, resulting in a complete cessation of the pleural effusion. Apart from the first two weeks our patient was treated on an out-patient basis for nearly six months.

**Conclusion:**

In this case of chylothorax caused by chronic lymphocytic leukemia, immunochemotherapy in combination with conservative treatment, and even consecutive radiotherapy, were not able to stop pleural effusion, despite the very good clinical response of the chronic lymphocytic leukemia to treatment.

Out-patient management using repetitive thoracocenteses can be safe as bridging until definitive surgical ligation of the thoracic duct.

## Introduction

Chylothorax is a rare condition defined by chyle entering the pleural space, caused by a disruption or blockade of the thoracic duct [1]. The pleural effusion is usually of milky white appearance due to a high lipid concentration. To distinguish chylothorax from nonchylous effusions, such as pseudochylothorax, the triglyceride level is determined. A triglyceride level greater than 110 mg/dL is highly suggestive of a chylous effusion. In cases where triglycerides range between 50 mg/dL and 110 mg/dL, a diagnosis of chylothorax can be made using lipid electrophoresis to detect the presence of chylomicrons [2,3].

Disruption of the thoracic duct may be due to traumatic or non-traumatic causes. In adults, the incidence of non-traumatic causes is reported between 50% and 70% of cases [4,5]. Among the non-traumatic causes, lymphoma and metastatic cancer are most common. Chronic lymphocytic leukemia (CLL), however, is a rare cause of chylothorax, with only a few cases reported in the literature so far [6].

Since chylothorax is an overall infrequent condition the best therapeutic approach is still under debate [1,5,7,8]. In particular, the use of modern immunochemotherapy, including the anti-CD20 antibody rituximab, in lymphoma-associated chylothorax has not yet been described.

Here we present the course of a patient presenting with chylothorax caused by a concomitantly diagnosed CLL who received conservative treatment in conjunction with immunochemotherapy, followed by radiotherapy and finally surgery to control his persisting pleural effusions.

## Case presentation

A 65-year-old male Caucasian patient was admitted with respiratory distress and suspected non-Hodgkin's lymphoma. His medical history revealed arterial hypertension, diabetes mellitus type 2 and cholecystolithiasis.

On clinical examination our obese patient (body mass index 41.5) suffered from dyspnea at rest. He presented with enlarged cervical and axillary lymph nodes, hepatosplenomegaly and diminished breath sounds over his right lung. A blood count revealed a leukocytosis of 35,000 leukocytes/μL of blood, with 80% partially atypical small lymphocytes and Gumprecht's shadow cells. Hemoglobin concentration and thrombocytes were within normal range. Immunophenotyping revealed that 67% of leukocytes were CD19+ B-lymphocytes, with co-expression of CD5, CD20, CD23 and a clonal restriction for the lambda light chain. This established the diagnosis of CLL. Bone marrow puncture demonstrated a medium degree of infiltration, with monoclonal B-cells beginning to replace the normal hematopoiesis.

**Figure 1:**
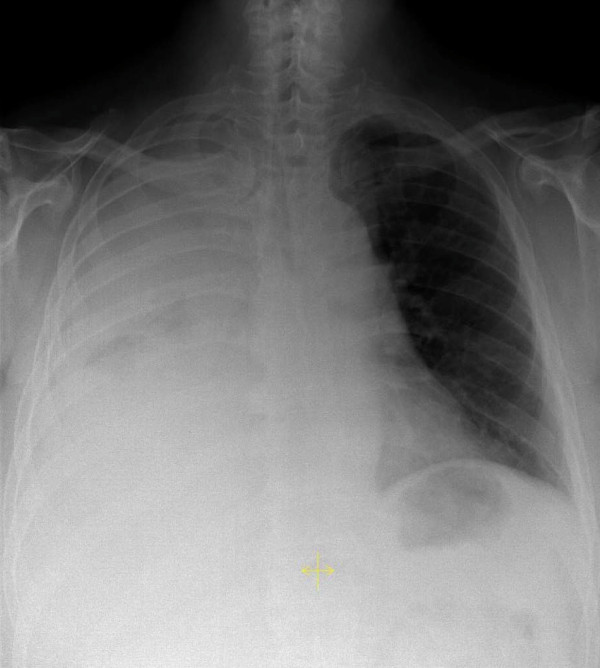
**Initial posterior-anterior chest X-ray demonstrating a complete right-sided opacity, later diagnosed as chylothorax**.

**Figure 2:**
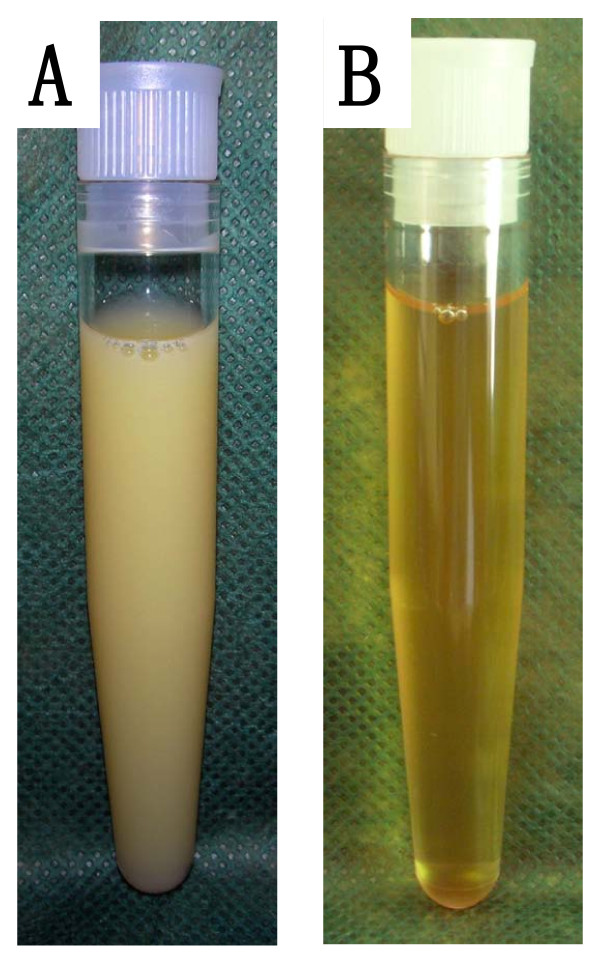
**Appearance of the pleural fluid before and after a low dietary fat intake**. **(A) **The high triglyceride content of over 700 mg/dL caused a milky appearance, characteristic of chylothorax. **(B) **A low-fat diet and concomitant reduced triglyceride levels in the pleural effusion resulted in a change towards a clear amber-colored fluid.

A chest X-ray was performed and showed a right-sided complete opacity suggesting a pleural effusion (Figure 1). Thoracentesis produced a milky pleural fluid (Figure 2A). The cellular content consisted of 80% lymphocytes, two thirds of which expressed the B-CLL phenotype. Further analysis of the pleural fluid revealed triglyceride levels over 700 mg/dL and cholesterol levels below 70 mg/dL, establishing the diagnosis of chylothorax. Our patient received a pleural drainage, which initially produced nearly 3 liters of chyle per 24 hours. A computed tomography (CT) scan depicted enlarged lymph nodes in the cervical, axillary and mediastinal region, and suspected splenic involvement with several hypodense lesions. Taking the findings into account, our patient was diagnosed with a right-sided chylothorax caused by a concomitantly diagnosed CLL, stage Binet B or Rai II.

The chylothorax represented a major complication of the CLL, and so immunochemotherapy consisting of fludarabine (25 mg/m^2 ^on days one to three), cyclophosphamide (250 mg/m^2 ^on days one to three) and rituximab (375 mg/m^2 ^on day one) was initiated. Our patient received four courses, repeated every four weeks. Since the therapeutic effect of reduced dietary intake on chylothorax had been described previously, our patient received total parenteral nutrition for two weeks, starting with the first cycle of the immunochemotherapy. The chylous effusion disappeared nearly completely, and the chest drain could be removed after 10 days. After two weeks an enteral low-fat diet enriched with medium-chain triglycerides was started, to continue therapy on an out-patient basis. Unfortunately the chylothorax relapsed and thoracentesis of a volume of 1 L to 1.5 L once to twice a week became necessary. Due to the low-fat intake the appearance of the pleural effusion had changed from milky-white to clear amber-colored (Figure 2B).

**Figure 3:**
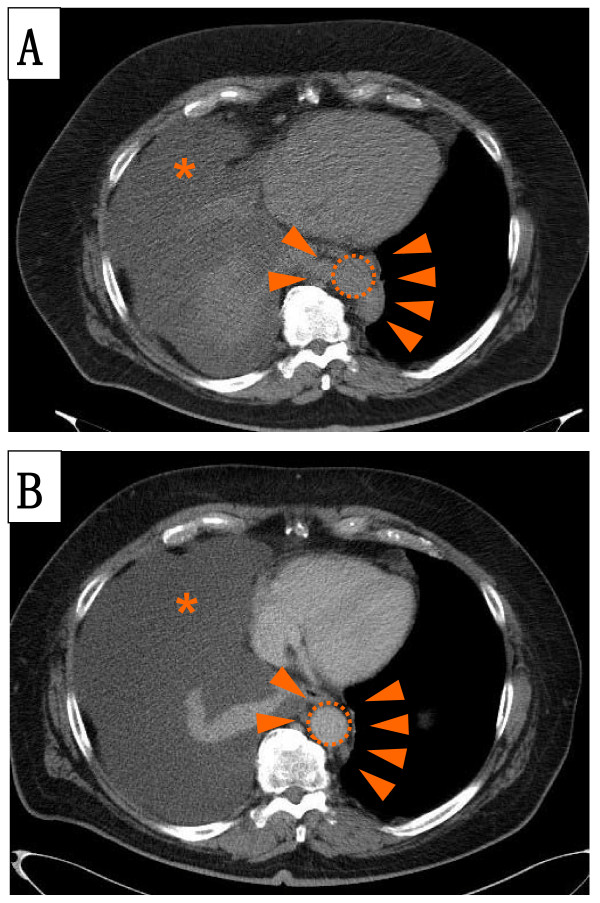
**A CT scan demonstrates para-aortal lymphadenopathy (A) before and (B) after two cycles of immunochemotherapy**. Para-aortal lymphadenopathy (arrow heads) might have been the most probable cause of the thoracic duct injury resulting in chylothorax (asterisk). Immunochemotherapy reduced the lymphadenopathy after only two cycles. Nevertheless, the pleural effusion (asterisk) still persisted, indicating that the thoracic duct injury had not healed. The aorta is indicated (dotted circle).

Our patient received four cycles of immunochemotherapy and regular thoracentesis on an out-patient basis. Since patients with protracted chylothorax are at risk of malnutrition and immunosuppression, our patient received antifungal and antiviral prophylaxis in addition to vitamin supplementation. However, the chylothorax persisted, despite a good clinical response of the CLL, with normalized blood counts and complete regression of the lymphadenopathy (Figure 3).

In light of this, percutaneous radiotherapy of his mediastinum and thoracic duct, with an overall dose of 24 Gy, was initiated. Radiation induces an inflammatory response which can result in an obliteration of the disrupted thoracic duct [9]. However, up to eight weeks after completion of the radiotherapy the chylothorax still persisted with continued requirement for regular pleural tapping.

Finally our patient agreed to a surgical intervention. A supradiaphragmal ligation of the thoracic duct via a right muscle sparing thoracotomy was carried out. In addition, a decortication of his right lung was necessary because, during his surgery, a pleural fibrosis was diagnosed. The pleural fibrosis was most likely caused by the long-term chylothorax with repetitive thoracenteses. Our patient quickly recovered and the pleural effusions ceased completely. The time from the first diagnosis of chylothorax until the final surgical intervention was six months. Our patient is still in complete remission after 24 months of follow-up.

## Discussion

Our patient presented with pronounced dyspnea, which was caused by a right-sided pleural effusion diagnosed as chylothorax. Pleural effusion, although not uncommon in non-Hodgkin's lymphoma, is less often seen in CLL [10]. The differential diagnosis of pleural effusion in a patient with CLL includes infection, pleural involvement and lymphatic obstruction [10]. Chylothorax, however, is a rare complication of non-Hodgkin's lymphoma (especially CLL) and should be considered by analyzing triglyceride and cholesterol concentrations in addition to routine parameters [3]. The present case showed that the chyle contained 80% B-CLL cells on immunophenotyping, replacing the normally present T-cells. This is in accordance with findings of two previous cases reported by Doerr *et al. *and Zimhony *et al. *[11,12], whereas Rice *et al. *found predominantly T-cells in the chyle of their CLL patient [6]. Therefore immunophenotyping of chyle may have limited value in diagnosing chylothorax in CLL patients.

Despite numerous anatomic variations, the thoracic duct usually arises from the cisterna chyli. From there it ascends through the aortic hiatus on the right side of the vertebral column and crosses to the left side between the sixth and fourth thoracic vertebra, before it empties into the junction of the left jugular and subclavian veins [5,7]. A right-sided chylothorax, therefore, indicates an injury below the fifth thoracic vertebra, which has to be taken into account when planning radiotherapy or surgery.

As depicted in Figure 2A the pleural fluid was initially milky white, which might already raise suspicion of its origin. However, the gross appearance of the chylothorax may be misleading in over half of the cases, since nutrition has a strong influence on lipid content and therefore on the appearance of the chyle [2,3]. With a low dietary fat intake the chyle clears to a serous appearance, as demonstrated in Figure 2B.

The amount of chyle produced per day correlates with dietary fat intake and was reported to range from 10 mL/kg to over 100 mL/kg body weight [8]. Due to this high amount of over 2 L on average per day, a rupture of the thoracic duct can result in rapid development of extensive pleural effusion with consecutive impaired breathing [1]. Therefore, immediate thoracentesis and a pleural drainage may be necessary. In addition, diet therapy, especially total parenteral nutrition in combination with pleural drainage, has been shown to be able to reduce chyle production and resolve chylothorax without increasing mortality [13]. In our patient, total parenteral nutrition resulted in a dramatic decrease in drained chyle from nearly 3 L to less than 100 mL per 24 hours and the pleural drainage could be removed after 10 days. However, after initiation of a low fat oral diet, the chylothorax recommenced, albeit at a lower rate, and required regular thoracenteses over the next three months.

In early reports, the mortality of chylothorax reached 50% for traumatic chylothorax and was fatal in non-traumatic cases [14]. This has now been reduced to a mortality of around 10% [1,7]. The amount of drained chyle in our patient was about 3 L per week, less than 500 mL per day, and so the risk of conservative treatment to evaluate the benefit of the anti-CD20 antibody, rituximab, in combination with immunochemotherapy seemed acceptable. Furthermore, it has been suggested that malignant chylothorax may not benefit from surgical intervention [15]. However, the combined approach of conservative treatment with a low fat diet and immunochemotherapy had no measurable effect on the chylothorax in this case.

Radiation may cause damage to the thoracic duct and induce chylothorax, most likely by inducing inflammation and obstruction [5]. On the other hand, the same mechanisms of radiation can be exploited to treat chylothorax [9,16]. In the present case however, radiation therapy had no effect, despite the prior effective anti-CLL therapy. A similar observation was reported by Zimhony *et al. *[12]. The chylothorax of their patient with CLL did not improve after chemotherapy and mediastinal irradiation, and required pleurodesis to resolve pleural effusion. However, mediastinal irradiation can be effective in CLL-associated chylothorax, as demonstrated by Ampil *et al.*, who reported the case of a female CLL patient who developed chylothorax under continuous treatment with chlorambucil and prednisone. Following mediastinal irradiation with 1000 cGy over five days, her chylous effusion resolved during the nearly five years of follow-up [17].

In our case, however, it was only surgical intervention that was able to stop the chyle effusion. One other case of CLL-associated chylothorax reported in the literature had also received successful ligation of the thoracic duct, albeit in combination with pleurodesis [11]. Despite her age of 93 years, that patient recovered well after surgery, indicating that thoracic duct ligation is well tolerated. Surgical ligation of the thoracic duct was introduced in 1946 by Lampson [14]. Modern, less traumatic surgery, such as muscle sparing thoracotomy as described by Bethencourt, allows a quick recovery and discharge of the patient [18]. Surgical ligation of the thoracic duct therefore seems a well tolerated therapeutic option in non-traumatic chylothorax also.

Another therapeutic option for chylothorax is pleurodesis. Mares and colleagues reported a case series of talc pleurodesis for chylothorax caused by lymphoma, including one patient with CLL and colon carcinoma. In contrast to our case, the patients in their series had end-stage lymphoma. Although the CLL patient was not specifically pointed out, pleurodesis was described as successful in all cases. However, high short-term mortality due to the underlying disease was noted [19]. Similarly the patient with CLL reported by Rice *et al*., who was treated symptomatically by repeated thoracentesis and total parenteral nutrition, died shortly after developing chylothorax [6]. The patient with CLL reported by Aranda *et al.*, who was started on chlorambucil and prednisone for CLL treatment and repeated thoracentesis after developing chylothorax, died shortly thereafter [20]. This indicates that CLL patients developing chylothorax late in their disease course may have a limited prognosis. Whether the prognosis for these patients might improve with modern immunochemotherapy remains to be seen. It is also interesting to note that patients with longer reported survival had either successful thoracic duct ligation, mediastinal irradiation or pleurodesis [11,12,17]. In our opinion, this allows for the conclusion that treating physicians should aim for definitive resolution of the chylothorax.

## Conclusion

Pleural drainage and total parenteral nutrition were efficient for initial emergency treatment of chylothorax caused by CLL. Addition of the anti-CD20 antibody rituximab to the chemotherapy was effective as anti-CLL therapy, but had no effect on the chylothorax. Whether this was due to individual features of this case, or may represent more general characteristics, remains to be seen. Under anti-infectious prophylaxis, regular surveillance and a chyle production of less than 500 mL per day, a prolonged treatment on an out-patient basis with regular thoracenteses was safe as a bridging treatment before definitive surgical intervention. Interdisciplinary case management of lymphoma-associated chylothorax, including hematologists, radiation oncologists and thoracic surgeons is desirable.

## Consent

Written informed consent was obtained from the patient for publication of this case report and any accompanying images. A copy of the written consent is available for review by the Editor-in-Chief of this journal

## Competing interests

The authors declare that they have no competing interests.

## Authors' contributions

All authors were directly involved in the care of the patient described in this case report. GAS, AM and BMS were responsible for the oncological care of the patient. HS performed the surgery. SS was responsible for application of the radiation therapy and KA reviewed the radiological diagnostics. GAS and BMS wrote the manuscript.
